# Cord blood methylation at TNFRSF17 is associated with early allergic phenotypes

**DOI:** 10.1007/s12026-024-09524-2

**Published:** 2024-07-31

**Authors:** Hanna Danielewicz, Artur Gurgul, Anna Dębińska, Anna Drabik-Chamerska, Lidia Hirnle, Andrzej Boznański

**Affiliations:** 1 1st Clinical Department of Pediatrics, Allergology and Cardiology, Wroclaw Medical University, Ul. Chałubińskiego 2a, 50-368 Wrocław, Poland; 2 Center for Experimental and Innovative Medicine, University of Agriculture in Krakow, Rędzina 1C, 30-248 Kraków, Poland; 3 1st Clinical Department of Gynecology and Obstetrics, Wroclaw Medical University, Ul. Chałubińskiego 5, 50-368 Wroclaw, Poland

**Keywords:** DNA methylation, Gene expression, Cord blood, Atopy, Maternal effect, EWAS, Epigenetics, Developmental programming

## Abstract

Food allergy and eczema are the earliest allergic phenotypes in childhood. These diseases could be related to either IgE-mediated or non-IgE-mediated reactions to the allergen. TNFRSF17 is a key molecule in B cell maturation and is important in both types of responses.

We conducted a study comparing the relative expression and the methylation status at the TNFRSF17 in regard to the child’s early atopic sensitisation and allergic phenotypes.

In the recruited population of 200 women and 174 children with available clinical data (physical examination by allergist and antigen-specific IgE measurements), 78 cord blood samples were included in the gene expression analysis (relative gene expression with GAPDH as reference by RT-PCR) and 96 samples with microarray DNA methylation data (whole genome methylation profile Infinium MethylationEPIC).

The altered TNFRSF17 methylation pattern in the cord blood at both single cg04453550 and mean methylation at upstream of TNFRSF17 was observed in children who developed food allergy and/or eczema in early childhood. The change in methylation profile was mirrored by the relative expression. The profile of IgE sensitisation to food and/or inhalant allergens was not significantly associated with either methylation or expression of TNFRSF17.

In conclusion, methylation at the upstream sites at TNFRSF17 in the cord blood at birth is associated with food allergy and eczema early in childhood.

**Supplementary Information:**

The online version contains supplementary material available at 10.1007/s12026-024-09524-2.

## Introduction

Food allergy and eczema are the first and earliest manifestations of allergic constitution. Through the life course, this manifestation transforms to asthma and/or allergic rhinitis. The sequence of events known as the allergic march, is one of most constant observations in the natural history of the allergic predisposition [1]. As a first manifestation, food allergy and eczema are interesting in regard to the plausible mechanism that leads to disease development by IgE and non-IgE-mediated responses. Since the incidence of food allergy at the population level is increasing menacingly in the last several years, the estimation of disease natural history is particularly important [2] [3].

Maternal atopy is a known risk factor for a child’s atopy, for both genetic and environmental impacts. The intrauterine environment, pro-allergic cytokines and different cell types influence child’s disease susceptibility [4]. Environmental effect is visible throughout the epigenetic changes with consequent gene silencing or activation. The final effect depends on the location of the CpG site. Those localised in the promoter typically are related to gene silencing if hypermethylated and gene activation whilst hypomethylated. Within the gene body, the effect could be both up or down regulation of gene expression [5] [6].

In our recent study, we have presented the methylation pattern of cord blood related to maternal atopy. One of the most significant genes within that analysis was TNFSFR17, which showed hypermethylation in the cord blood of infants form atopic mothers [7]. In the current analysis, we first compared the relative expression of TNFRSF17 in cord blood of children form atopic and non-atopic mothers and further compared this parameter in relation to the child’s atopy and clinical phenotypes at early childhood, hypothesising that alleviated methylation of TNFRSF17 in cord blood could predict allergic phenotype in early childhood, and further researching the impact of maternal atopy on child’s sensitisation status.

## Results

The methylation data, allergen-specific IgE data, clinical data, and relative expression data were available for *n* = 96, 91, 94, and 78 children, respectively. Atopic sensitisation was diagnosed in 18% of children, and infant eczema and/or food allergy was diagnosed in 52% of children at 12–18 months of life. The characteristics of the study group are presented in Table 1.

**Table 1:** Characteristics of the study group (*n* = 174)

Parental and environmental factors	
Maternal atopy (sIgE)	82 (47.13%)
Maternal age	29.09 ± 2.7
GWG	14.19 ± 4.51
Paternal atopy (self-reported)	52 (29.89%)
Pets at home	55 (31.60%)
Child’s factors	
Gestational age	39.525 ± 1.29
Birth weight	3446.06 ± 492.98
Natural birth	86 (49.43%)
C-section	88 (50.57%)
Sex-female	70 (40.23%)
Sex-male	104 (59.77%)
Atopic sensitisation	33 (18.96%)
Food allergy	60 (34.48%)
Food allergy and/or eczema	92 (52.87%)

**Fig. 1:**
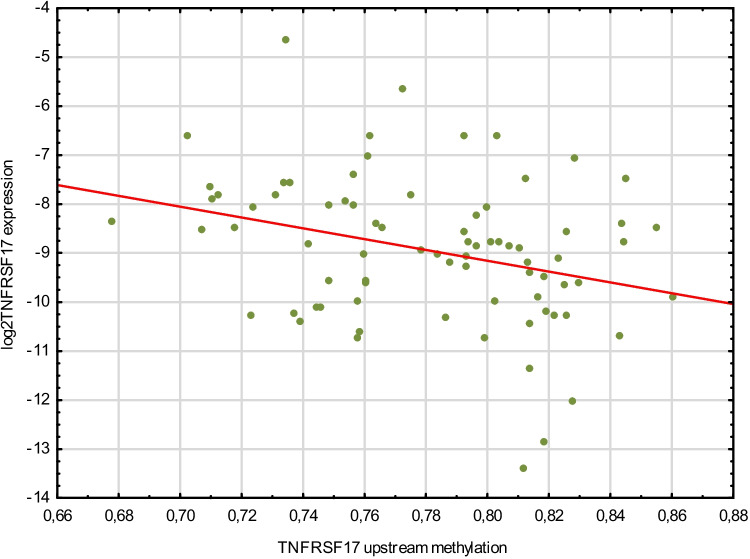
Scatter plot illustrating the relationship between mean methylation beta at the upstream sites at TNFRSF17 and log2 transformed concentration ratio of mRNA for TNFRSF17 normalised to GAPDH as the reference (*r* =  − 0.31 and *p* = 0.0057)

Relative gene expression analysis of TNFSFR17 showed a significant correlation with the methylation data, Spearman *r* = 0.3, with the reverse pattern—the higher the methylation, the lower the gene expression, which is consistent with the observation for other genes and methylation in the promoter sites (Fig. 1).

**Fig. 2:**
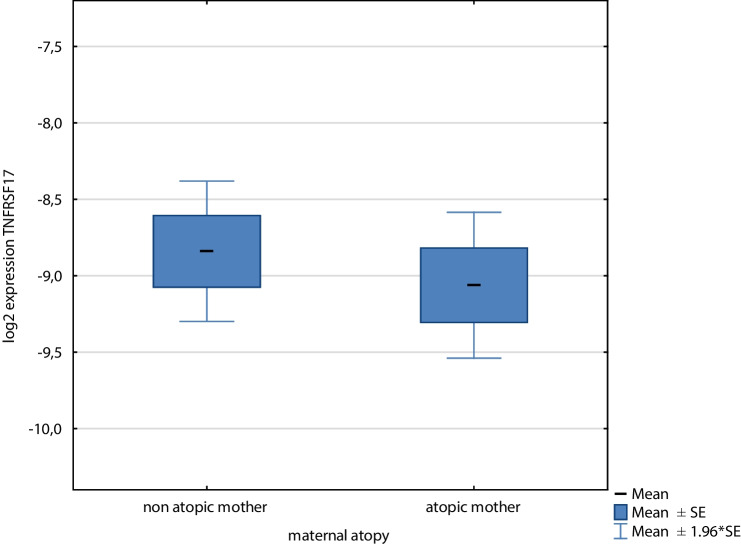
The mean values of TNFRSF17 log2 expression transformed concentration ratio, normalised to GAPDH as the reference, in the group of children born to atopic and non-atopic mothers

However, we have not shown a significant association for maternal atopy and TNFRSF17 expression. The trend was visible, with a lower expression in the maternal atopic group (Fig. 2).

Furthermore, we analysed the clinical data of children—both allergen specific IgE as estimates of atopy and clinical diagnosis of primary allergic outcomes such as infant eczema and food allergy in relation to methylation at cg04453550 TNFRSF17, mean methylation at the upstream region of TNFRSF17, and expression data.

**Fig. 3:**
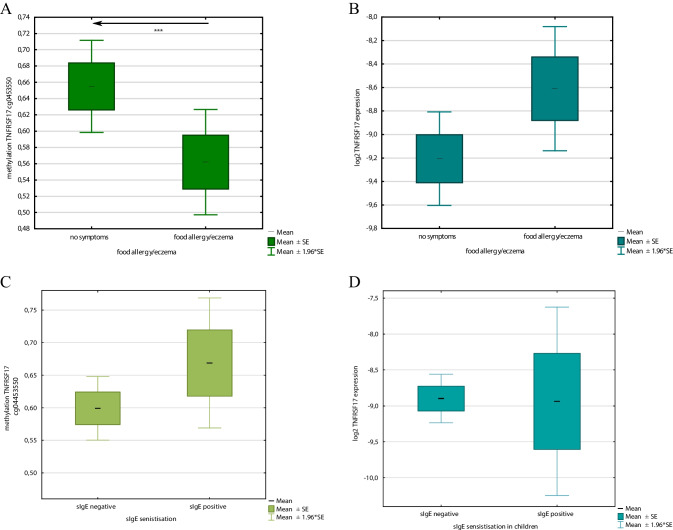
**A** The mean values of DNA methylation beta at cg04453550 within TNFRSF17 assigned as having food allergy or/and eczema or without any allergic phenotype based on the clinical diagnosis at 12–18 months. The differences were statistically significant, UMW test, *p* < 0.05. **B** The mean values of TNFRSF17 log2 transformed concentration ratio, normalised to GAPDH as the reference, in the group of children assigned as having food allergy or/and eczema and without any allergic phenotype based on the clinical diagnosis at 12–18 months, UMW test.** C** The mean values of DNA methylation beta at cg04453550 within TNFRSF17 based on the sIgE results at 12–18 months. **D** The mean values of TNFRSF17 log2 transformed concentration ratio, normalised to GAPDH as the reference, in the group of children assigned based on the sIgE results (atopic sensitisation) at 12–18 months, UMW test, for both *p* > 0.05

We have observed significant association for DNA methylation at TNFRSF17 and infant eczema and/or food allergy (Fig. 3A), but not atopic sensitisation (Fig. 3C). We also did not achieve significant results for the gene expression of TNFRSF17 for both food allergy/eczema diagnosis (Fig. 3B) and children’s atopic sensitisation (Fig. 3D). In the regression analysis, DNA methylation of TNFSFR17 was an independent factor influencing the appearance of the allergic phenotype at 12–18 months in a model including maternal atopy, paternal atopy, having pets at home during pregnancy, C-section, GWG and sex as covariates (Tables 2 and 3). Children with food allergy and/or eczema presented lower methylation for TNFRSF17. We did not observe a significant relationship for the TNFRSF17 gene expression and these outcomes. In the subgroup analysis for methylation/expression and single factors we observed some differences in TNFSRF17 methylation in regard to sex, C section, maternal atopy and having pets at home, thus suggesting individual impact of these features on the outcome (Supplementary Table 1).

**Table 2:** Logistic regression model for atopic sensitisation and having food allergy and/or eczema at 12–18 months of life, with covariates such as maternal and paternal atopy, having pets at home during pregnancy, C section GWG, and sex (*n* = 174); no predictor was significant in the model

	Atopic sensitisation		Food allergy/eczema	
	OR	95% CI	OR	95% CI
Maternal atopy (sIgE)	1.88	0.82–4.29	1.07	0.57–2.00
Paternal atopy (self-reported)	1.65	0.72–3.79	1.29	0.66–2.51
Pets at home	1.31	0.55–3.11	0.81	0.42–1.56
C-section	0.55	0.25–1.25	1.26	0.68–2.34
GWG	0.99	0.91–1.08	0.98	0.91–1.04
Sex—male	0.46	0.21–1.05	1.13	0.66–2.12

**Table 3:** Logistic regression model for having food allergy and/or eczema at 12–18 months of life, with covariates such as maternal and paternal atopy, having pets at home during pregnancy, C section, GWG, and sex. **A** log2 transformed concentration ratio for mRNA of TNFRSF17, normalised to GAPDH as the reference, **B** *M* value for DNA methylation of cg04453550, and **C** with use of *M* value for mean methylation at the upstream sites within TNFRSF17

Food allergy/eczema								
*n* = 78	OR	95% CI	*n* = 94	OR	95% CI	*n* = 94	OR	95% CI
A Log2TNFRSF17 expression	1.33	0.90–1.99	B M methylation at TNFRSF17 cg04453550	0.72	0.52–0.98	C *M* mean methylation at upstream TNFRSF17	0.13	0.03–0.5
Maternal atopy (sIgE)	1.34	0.49–3.65		1.30	0.5–3.4		1.21	0.46–3.13
Paternal atopy (self-reported)	1.37	0.43–4.35		1.44	0.52–3.98		1.35	0.47–3.82
Pets at home	0.56	0.2–1.63		0.51	0.19–1.35		0.48	0.18–1.3
C-section	0.83	2.4–0.28		0.83	0.29–2.41		1.05	0.42–2.59
GWG	0.96	0.86–1.0		0.98	0.89–1.07		0.98	0.89–1.08
Sex -male	1.08	0.39–2.96		1.30	0.53–3.2		1.44	0.57–3.67

## Discussion

We revealed that DNA methylation of TNFRSF17 is changed at birth in children developing food allergy and/or eczema at 12–18 months of life, with lower methylation in the food allergy/eczema group. For the expression analysis neither maternal atopy nor child’s sIgE sensitisation or food allergy/eczema showed significant relationship, however trend was visible consistent with the direction of changes in the DNA methylation.

TNFRSF17 also known as BCMA (other aliases BCM, TNFRSF13a, and CD269) is a member of the TRAF family. It is preferentially expressed in mature B lymphocytes and may be important for B cell development and the allergic response. This receptor has been shown to specifically bind to TNF13b (BAFF). BAFF is produced by both lymphoid and non-lymphoid cells, including bronchial and nasal epithelial cells. The levels of BAFF have been revealed to be upregulated by INF gamma, IL10, and CD40—molecules produced during inflammation. BAFF is an important regulator of B cell survival, maturation, and immunoglobulin class–switch recombination. It is also an important regulator of T cell mediated responses. In a mouse model, binding BAFF to Th0 lymphocytes enhances Th1 activity. BAFF could also affect the function and proliferation of Th17 cells engaged in non-IgE-mediated asthma and food allergy. Increased serum BAFF levels have been observed in both conditions. Higher levels are also present in non-atopic compared with atopic subjects with no correlation between IgE level and BAFF. Possibly at gut level BAFF is produced locally as a response to inflammation, resulting in both—local immunoglobulin production and T cell activation [8]. BAFF also shares some properties with PAF—another factor associated with non-IgE-mediated allergic reactions [9].

TNFRSF17 is associated mainly with B cell maturation. In the mouse model or allergic airway inflammation, increased BCMA expression was observed in pre-B and pro-B cells, with the further rise after allergen challenge. Together with increased BAFF levels in severe asthmatic, this suggests important role of both molecules in allergic inflammation [10] [11]. The role of BAFF and its receptors has been also indicated in other studies regarding asthma, food allergy and rhinosinusitis and nasal polyps [12, 12, 13]. Soluble forms of BCMA have been found to decrease in hypogammaglobulinemia and increase in myeloma [14]. Increased expression has been observed in adolescence with single food allergy (peanut) in comparison to multi-food allergy individuals [15].

However, the exact role of BAFF/BCMA in allergy remains unclear. Apart from connection to immunoglobulin switching and antigen-specific IgE production and IgE memory, there seems to be other mechanism responsible specifically for non-IgE response and Breg function [12].

B reg could induce Treg and supress inflammation in atopic dermatitis and asthma and also supress food-allergy related intestine inflammation. Breg maturation goes through TNFRSF17 and APRIL system, with the differentiation of naïve B cells to IgA + IL10-producing B cells [16].

TNFRSF17 could also bind to other TRAF family members, making it responsible for different cell survival and proliferation. Expression studies of different human tissue showed a higher expression of this molecule in the gastrointestinal tract and lymph nodes, which suggest the key role in immune responses and food allergies [17]. Within the family, different receptors have specific functions. Some of them are related to TGF beta, NF kappa, and JNK pathways, which are important factors in allergy development [18]. Specifically, TNFSFR14s is associated with allergic rhinitis. The plausible mechanism for this association is related to T cell differentiation and the subsequent changes in IL13, IFN gamma, and TGF beta [19].

Epigenetic studies regarding food allergy in early childhood are scarce. An association of TNFRSF17 with IgE-mediated food allergy has been revealed in study regarding infants [20]. In this research, a pathway analysis of specific DMP-associated genes revealed an enrichment for “intestinal immune network for IgA production” in 12 months old infants with food allergy. The genes in this pathway that were differentially methylated included HLA-DQB1, CD80, and TNFRSF17 [20]. Other studies showed altered methylation in genes—such as INF gamma, IL-4, IL5, and FOXP3—as associated with cow milk allergy and miR-193a-5p presented with some evidence as downregulated in allergic children. There were also some hits for DHX58, ZNF281, EIF 42A, and HTRA2 [21].

Food allergy and eczema are believed to be the first manifestations of the allergic phenotype, which later in life develops as allergic rhinitis and asthma, following the allergic march schedule [22]. Infant eczema, as an early presentation of atopic dermatitis, is believed to precede the IgE-mediated food allergy with increased risk associated with the penetration of allergens through the damaged skin barrier [23]. 30–40% of patients with atopic dermatitis have a food allergy [2]. Eczema usually begins between 3 and 6 months with a bimodal age distribution. An increase is observed in infancy followed by a stable decline into childhood and early adulthood [24]. The mechanism is complex—mixed IgE-mediated and non-IgE-mediated. The first component depends on the Th2 response, with the release of cytokines such as IL-4, IL-13, and IL-21, which act through the STAT-JAK pathways. JAK inhibitors are used recently as therapeutic agents [25].

Food allergy seems to be an even more complex disorder with a spectrum of symptoms from different organs—gastrointestinal, skin through the respiratory system with high heterogeneity [26]. The mechanism is either IgE-mediated or non-IgE-mediated or mixed [27] [28]. It is estimated that in the first year of life, up to half of allergic reactions to food could be non-IgE-mediated and present both as infant eczema or gastrointestinal symptoms [2].

Non-IgE-mediated food allergy is believed to be cellular-dependent with a delayed response and a possible mechanism including IL10 and IL5 [29]. An inflammatory cytokine TNF-α has been shown to be overexpressed in this type of allergic reaction, whereas the TGF-β1 receptor activity and TGF-β ligand expression were shown to be decreased. It is hypothesised that abnormal expression of TNF-α and TGF-β weakens the epithelial barrier in the gut [30]. TNF alpha together with faecal calprotectin, alpha 1 antitrypsin beta defensin, EDN, and CP have been proposed as biomarkers of non-IgE-mediated food allergy [31]. Based on our results, we can propose TNFRSF17 as a new disease marker.

The prevalence of atopic sensitisation in small children is not well studied. The literature in that area is limited, and only few studies have focused on the subject. However, in the BEAT study (study enquiring about the early introduction of allergenic food), 10% of 4-month-old infants enrolled at screening were already sensitised to eggs and 8.7% of 12-month-old babies were sensitised to peanuts [32], which implies a high rate of food allergen sensitisation at this early age. Similarly, there is not much evidence for aeroallergen sensitisation within infancy. Some children are carefully evaluated if they have asthma symptoms, but still in clinical practise, it is believed that the IgE response is low and rarely detected with current technology. Some authors deal with that problem by regarding a lower threshold for allergen sensitisation of 0.2 or 0.1 kU/mL, instead of the widely accepted value of 0.35 kU/mL—which we used in our analysis.

There is not a good understanding of what is the meaning of an increased allergen specific IgE at that age. Analyses comparing allergen-specific IgE at birth and 12 months of life showed different patterns with a predominantly transient character of sensitisation at birth. However, the prevalence of sensitisation was astonishingly high in that population, where 44% of children were sensitised at 12 months with a cut-off of 0.2. Surprisingly, no association was shown for sIgE and the diagnosis of food allergy (which was 9.8%) and atopic eczema (2, 1%). On the other hand, this transient sensitisation was related to maternal food sensitisation [33]. Similarly, in another study, maternal atopy seemed to influence the IgE sensitisation pattern in children at birth, and this effect waned in time and was not present at the age of 12 months [34]. Also other birth cohorts were studied for the time-dependent changes of sIgE, since the first year of life to adulthood. Inversely, in comparison to transient changes in infancy, for sensitisation at 1 year, there was an increase in prevalence of sensitisation through the life course from 19% at 1 year up to 71% at 24 years. [35]

The results of our study indicate different pathways for sIgE sensitisation and food allergy/eczema in the first years of life, first with increased methylation at the upstream site of TNFRSF17 and the second with decreased. This is further confirmed by the concordance of the direction for the maternal and child’s atopy association with the methylation data. It also suggests that both food allergy and infant eczema at that age are mainly non-IgE-mediated.

There are few studies regarding the TNFRSF17 methylation status. Silencing the gene by hypermethylation was observed in classical Hodgkin’s lymphoma [36]. Changes in the expression were observed in the idiopathic pulmonary fibrosis, but not asthma [37]. In OVA-sensitised and challenged mice, BAFF (which biological function is mediated by TNFRSF17) expression is increased together with increased IgE production and bronchial thickening suggesting the key role in asthma pathogenesis [38].

The relationship between methylation and expression in our study was significant; however, the correlation coefficient at the level of 0.3 seems to be the threshold value for such an analysis and could be perceived as low. This suggests a more complex mechanism of the regulation of gene expression, which possibly relates not only to the change in one CpG site but also to some other regulating factors such as miRNA. A similar pattern, with only partial correlation between the methylation and expression data, was observed in other studies [15].

## Limitations

Our study has some strengths, such as connecting data between two types of analysis, whole genome (EWAS),and experimental (expression), together with clinical data for a larger group of participants. However, some limitation exists. The clinical diagnosis of eczema and food allergy is generally challenging. A great amount of heterogeneity in the diagnostic criteria has been shown in other studies [24]. We also pooled the patients diagnosed with food allergy and eczema because the majority of cases have both diagnoses, thus unfortunately increasing the heterogeneity. Another problem was the low prevalence of sIgE sensitisation, making the comparison between groups less powerful due to low numbers. It is possible in the future to use lower threshold to increase the sensitivity. Another limitation was the low gene expression profile from cord blood. Some samples were assigned as negative if Ct was high and were excluded from further analysis. More sensitive tests are required for such tests in the future studies.

## Conclusion

Methylation at the upstream sites at TNFRSF17 at birth is an independent factor affecting the prevalence of food allergy and eczema. Both are the first manifestations of allergy and precede the allergic march leading to asthma and allergic rhinitis later in life. These results are in a concordance with DOHaD (The Developmental Origins of Health and Disease) hypothesis and confirm the developmental programming of allergic diseases in utero.

Establishing the exact role that TNFRSF17 plays in the pathogenesis of early allergic phenotypes requires further studies.

## Methods

### Population

**Fig. 4:**
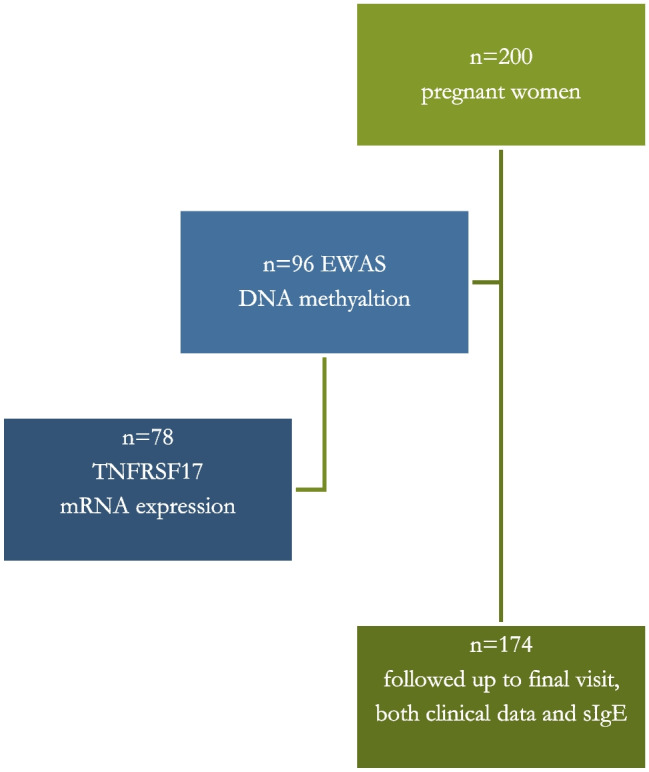
Study design diagram

Two hundred pregnant women in the third trimester of pregnancy were enrolled in the study. The inclusion criteria, study design, and characteristics of the mothers are described elsewhere [39]. One hundred seventy-four children were followed up to the final visit at 12–18 months, with data available for both sIgE measurements and clinical evaluations by an allergy specialist at 3, 6, and 12–18 months of life. Twenty-six children were lost at some point during the follow-up period. The diagram illustrating the study group is presented in Fig. 4.

### Relative gene expression

Ninety-six samples of cord blood were included in the gene expression analysis. The samples were collected into Tempus Blood RNA tubes containing a stabilizing reagent (Thermo Fisher Scientific) for RNA extraction. RNA was extracted from whole-blood samples using the QIAamp RNA kit (Qiagen Inc., Valencia, CA, USA). Reverse transcription to cDNA was carried out using the First Strand DNA Reverse Transcription Kit (Roche Ltd., Basel, Switzerland) according to the manufacturer’s protocol. The expression analysis was performed with the LighCycler 1.5 and specific TaqMan probes for TNFRSF17 (Thermo Fisher Scientific, Carlsbad, CA, USA 92008) Hs00171292_m1. And GAPDH—Hs99999905_m1. GAPDH was used as a reference for normalisation. The negative controls (including only primers) were included in each experiment. The concentration ratio of TNFRSF17:GAPDH, calculated by LightCyclcer Software 4.0, was presented as a measure of the relative gene expression. Samples that did not show expression were not included in the analysis. Eventually, the experimental data from 78 samples were available for analysis.

### Methylation profile

The whole genome analysis of the DNA methylation in cord blood with regard to maternal atopy is presented in detail in a previous publication [7]. Within TNFRSF17–7 CpG were analysed, amongst them, cg04453550 showed a high ranking according to the RnBead score and a high methylation difference. The RnBead score complies a statistical significance with the methylation difference and the size effect. For comparison purposes with the expression data, normalised beta (methylation) values were used, as detected using RnBeads software and Infinium MethylationEPIC arrays (Illumina).

Quality control was provided according to standard procedure, described in detail in our previous study [7]. In brief, intensities after scanning were added to quality control, filtering and normalisation. Initially control was performed using the BeadArray Reporter Software (Illumina). Infinium probes covered with SNPs, and sites with missing *β* values were detached. Sites and samples were filtered using a Greedy approach. The gotten data were normalised using the BMIQ procedure. The batch effects, such as array and array position as well as other hidden confounders, were recognised and removed using the surrogate variable analysis (SVA) method.

### Clinical evaluation

The diagnosis of food allergy was estimated on the basis of a parental report of the clinical diagnosis by a family doctor and an introduction of an elimination diet and/or skin or gastrointestinal symptoms consistent with food allergy and/or symptoms consistent with food allergy and a positive sIgE for food. The diagnosis of infant eczema was estimated either as reported by parents and/or observed during the clinical physical evaluation by an allergy specialist.

### Allergen-specific IgE measurement

The measurement of sIgE was performed from blood obtained at the last visit at 12–18 months. Children were assigned as being atopic if they presented at least one positive reaction (specific immunoglobulin E [sIgE] ≥ 0.35 kUL^−1^) to any of 20 allergens, including cow’s milk, egg, wheat flour, peanuts, dust mites, trees, grasses, cat, dog, and Alternaria and Cladosporium allergens (POLYCHECK, Biocheck, Münster, Germany).

### Statistical analysis

Statistical analysis was performed by Statistica 13.5. For the comparison of continues data such as methylation and expression in groups regarding allergic phenotypes, the Mann–Whitney *U* test was applied, and *p* values < 0.05 were regarded as statistically significant. For estimating probability of having atopic sensitisation and food allergy/eczema in regard to different dichotomous and continuous variables, logistic regression was applied. For regression analysis, beta values were transformed to *M* values by the equation *M* = log2(beta/(1-beta)).

The study was approved by the Ethical Committee of the Wroclaw Medical University and all participants signed informed consent forms, for children informed consent had been obtained from a parent.

All methods were performed in accordance with the relevant guidelines and regulations.

## Supplementary Information

Below is the link to the electronic supplementary material.

Supplementary file1 (DOCX 21 KB)

## Data Availability

The raw datasets generated during the current study are not publicly available due to confidential reasons. Can be provided under reasonable request.

## Abbreviations

DM: Differentially methylated
EWAS: Epigenome-wide association study
FDR: False discovery rate

## Declarations

### Disclaimer

The funders had no role in the design of the study; in the collection, analyses, or interpretation of data; in the writing of the manuscript; or in the decision to publish the results.
