# Supplementary material for: Cord blood methylation at TNFRSF17 is associated with early allergic phenotypes
Source: Immunol Res. 2024 Jul 31;72(6):1259–67. doi: 10.1007/s12026-024-09524-2 (PMC11618142; doi:10.1007/s12026-024-09524-2)
Supplement: Supplementary file 1 — Supplementary file1 (DOCX 21 KB) [file 12026_2024_9524_MOESM1_ESM.docx]

|  | FA/eczema yes  Mean (±SD) | FA/eczema no | P  Test UMW |
| --- | --- | --- | --- |
| ***TNFRSF17 expression*** |  |  |  |
| **C section** | **-7.694±1.09** | **-8.920±1.51** | **0.026** |
| Natural birth | -9.429±1.58 | -9.485±1.10 | ns |
| Boys | -8.619±1.54 | -8.980±1.09 | ns |
| **Girls** | **-8.591±1.83** | **-9.715±1.55** | **0.016** |
| Maternal atopy yes | -8.812±1.80 | -9.298±1.18 | ns |
| Maternal atopy no | -8.383±1.40 | -9.192±1.43 | ns |
| Paternal atopy yes | -8.181±1.55 | -8.978±1.93 | ns |
| Paternal atopy no | -8.774±1.64 | -9.315±1.11 | ns |
| Pets yes | -8.629±1.10 | -9.648±1.36 | ns |
| Pets no | -8.603±1.77 | -8.896±1.21 | ns |
| ***TNFRSF17 methylation***  ***a****t* cg04453550 |  |  |  |
| **C section** | **0.524±0.20** | **0.619±0.23** | **0.046** |
| Natural birth | 0.595±0.23 | 0.680±0.17 | ns |
| Boys | 0.599±0.22 | 0.647±0.21 | ns |
| **Girls** | **0.496±0.20** | **0.666±0.18** | **0.0067** |
| Maternal atopy yes | 0.628±0.18 | 0.717±0.17 | ns |
| Maternal atopy no | 0.488±0.23 | 0.587±0.21 | ns |
| Paternal atopy yes | 0.533±0.19 | 0.594±0.23 | ns |
| Paternal atopy no | 0.573±0.22 | 0.671±0.19 | ns |
| Pets yes | 0.548±0.21 | 0.619±0.216 | ns |
| **Pets no** | **0.566**±**0.22** | **0.566**±**0.19** | **0.0045** |
| ***TNFRSF17 Methylation at up stream*** |  |  |  |
| **C section** | **0.760±0.04** | **0.794±0.040** | **0.01** |
| Natural birth | 0.775±0.04 | 0.796±0.03 | ns |
| Boys | 0.776±0.045 | 0.794±0.037 | ns |
| **Girls** | **0.753±0.03** | **0.797±0.03** | **0.019** |
| **Maternal atopy yes** | **0.772±0.04** | **0.806±0.03** | **0.0075** |
| Maternal atopy no | 0.763±0.04 | 0.763±0.03 | ns |
| Paternal atopy yes | 0.767±0.04 | 0.780±0.03 | ns |
| **Paternal atopy no** | **0.768±0.04** | **0.799±0.03** | **0.0037** |
| Pets yes | 0.768±0.05 | 0.787±0.03 | ns |
| **Pets no** | **0.768±0.04** | **0.801±0.03** | **0.0038** |

Supplementary Table 1 Subsamples analysis for TNFRSF17 expression (log2 transformed concentration ratio for mRNA of TNFRSF17. normalised to GAPDH as the reference). DNA methylation at cg04453550 and DNA methylation at the upstream sites within TNFRSF17. UMW test. p < 0.05. was considered significant.
